# Association Between the Severity of Diabetic Retinopathy and Optical Coherence Tomography Angiography Metrics

**DOI:** 10.3389/fendo.2021.777552

**Published:** 2021-12-10

**Authors:** Binxin Xu, Jiahui Chen, Shaohua Zhang, Shengli Shen, Xuan Lan, Zhineng Chen, Zhiqiang Yan, Bingxiang Xu

**Affiliations:** ^1^ Human Phenome Institute, State Key Laboratory of Medical Neurobiology and MOE Frontiers Center for Brain Science, Institute of Brain Science, School of Life Sciences, Fudan University, Shanghai, China; ^2^ Institute of Molecular Physiology, Shenzhen Bay Laboratory, Shenzhen, China; ^3^ Department of Ophthalmology and Vision Science, Eye and ENT Hospital of Fudan University, Shanghai, China; ^4^ NHC Key Laboratory of Myopia (Fudan University), Laboratory of Myopia, Chinese Academy of Medical Sciences, Shanghai, China; ^5^ Key Laboratory of Visual Impairment and Restoration of Shanghai, Shanghai, China; ^6^ Human Phenome Institute, Fudan University, Shanghai, China; ^7^ School of Computer Science, Fudan University, Shanghai, China; ^8^ School of Kinesiology, Shanghai University of Sport, Shanghai, China; ^9^ State Key Laboratory of Translational Medicine and Innovative Drug Development, Jiangsu Simcere Diagnostics Co., Ltd., Nanjing, China; ^10^ Nanjing Simcere Medical Laboratory Science Co., Ltd., Nanjing, China

**Keywords:** diabetic retinopathy, diabetes, optical coherence tomography angiography, retinal vessel segmentation, retinal vessel quantification, systemic risk factors

## Abstract

Diabetic retinopathy, the most serious ocular complication of diabetes, imposes a serious economic burden on society. Automatic and objective assessment of vessel changes can effectively manage diabetic retinopathy and prevent blindness. Optical coherence tomography angiography (OCTA) metrics have been confirmed to be used to assess vessel changes. The accuracy and reliability of OCTA metrics are restricted by vessel segmentation methods. In this study, a multi-branch retinal vessel segmentation method is proposed, which is comparable to the segmentation results obtained from the manual segmentation, effectively extracting vessels in low contrast areas and improving the integrity of the extracted vessels. OCTA metrics based on the proposed segmentation method were validated to be reliable for further analysis of the relationship between OCTA metrics and diabetes and the severity of diabetic retinopathy. Changes in vessel morphology are influenced by systemic risk factors. However, there is a lack of analysis of the relationship between OCTA metrics and systemic risk factors. We conducted a cross-sectional study that included 362 eyes of 221 diabetic patients and 1,151 eyes of 587 healthy people. Eight systemic risk factors were confirmed to be closely related to diabetes. After controlling these systemic risk factors, significant OCTA metrics (such as vessel complexity index, vessel diameter index, and mean thickness of retinal nerve fiber layer centered in the macular) were found to be related to diabetic retinopathy and severe diabetic retinopathy. This study provides evidence to support the potential value of OCTA metrics as biomarkers of diabetic retinopathy.

## Introduction

Diabetic retinopathy (DR), is one of the most frequent causes of blindness in the working-age population (1). DR causes expression of vascular endothelial growth factor (2), inflammation (3), upregulation of renin–angiotensin (4), oxidative stress (5), activation of protein kinase C (6), the formation of sorbitol (7), and advanced glycation end-products (8), which lead to an increased permeability of retinal vessel, capillary non-perfusion area, microaneurysm formation, and retinal endothelial cell damage. DR also causes retinal neurodegeneration (9, 10) and predicts an increased risk of life-threatening systemic vascular complications (11).

Optical coherence tomography (OCT) is a new non-contact and non-invasive imaging technique. Combined with the optical coherence tomography angiography (OCTA), it allows a layered view of the vascular morphology and blood flow alterations of the retina and choroid, enabling a more in-depth study of specific retinal capillary layer lesions caused by DR (12). Several studies have reported that OCTA metrics were associated with the severity of DR, such as foveal avascular zone (FAZ) area and vessel density, indicating the potential value of OCTA for managing DR progress (13–15). Vessel segmentation is a necessary step for quantifying OCTA metrics (such as vessel density, vessel skeleton fractal dimension, and vessel skeleton density). However, the retinal vessel segmentation methods used in these studies have two glaring limitations: i) discontinuity of segmented vessels, and ii) poor and false segmentation of low-contrast vessel regions.

Most published OCTA-based retinal vessel segmentation methods can be divided into the following kinds: local adaptive thresholds, hessian filter, and non-local means filter. Reif et al. (16) combined the low-pass filter and the local adaptive threshold to achieve the segmentation of vascular. Chu et al. (17) used the global threshold to remove noise, the hessian matrix to enhance the image, and the adaptive threshold to segment blood vessels. The two methods previously mentioned have low complexity and can achieve available segmentation results, but their anti-noise ability is poor. The segmentation method implemented by Tang et al. (14) prevented the enhancement of noise by a non-local means filter and segmented vessels by using a phansalkar adaptive local thresholding. However, the method proposed by Tang et al. (14) has high computational complexity and is prone to introduce false-positive results. Due to the amount of noise present in the OCTA images, the poor quality of the capillaries, the requirement for continuity of the segmented vessels, and the existing vessel segmentation methods do not achieve satisfactory results.

In response to the above limitations, this study developed a multi-branch retinal vessel segmentation method (abbreviated as MRVSM) that does not enhance noise and can effectively extract low-contrast capillaries. MRVSM designed special methods and corresponding parameters to segment the large vessels and capillaries separately since the pixel intensities vary considerably between vessels.

Reviewing the previous studies on the relationship between OCTA metrics and the severity of DR, a comprehensive analysis of the effect of systemic risk factors on OCTA metrics is still lacking. Consideration of systemic risk factors is crucial for the reliable study of the relationship between the OCTA metrics and the severity of DR. In this study, we analyzed the relationship of OCTA metrics to 36 systemic risk factors. After controlling the identified systemic risk factors, we studied whether the OCTA metrics were associated with DR and severe DR. This study can identify eyes at high risk of DR progression, which facilitates early diagnosis and timely intervention for DR.

## Materials and Methods

### Participants

The study followed the ethical standards set out in the 1964 Declaration of Helsinki and was approved by the Ethics Committee of Eye and ENT Hospital of Fudan University and the Ethics Committee of School of Life Sciences of Fudan University with informed consent forms.

Approximately 596 healthy participants (1,192 eyes) were eligible for the recruitment criteria. Among them, 100 healthy participants were recruited from the Eye and ENT Hospital of Fudan University and 496 healthy participants were recruited from the Human Phenome Institute of Fudan University. Approximately 240 diabetes participants (418 eyes) were recruited from the Eye and ENT Hospital of Fudan University. After the image quality control, the study excluded 97 eyes (reasons shown in **Supplementary Figure 1**). Finally, the study included 587 healthy participants (1,151 eyes) and 221 diabetes participants (362 eyes). Approximately 54 healthy participants and 68 diabetes participants from the Eye and ENT Hospital of Fudan University and 496 from the Human Phenome Institute of Fudan University underwent systemic test, including the fasting blood test, urine test, blood pressure test, pulse test, vision test, and measurements of demographic variables. All the participants measured for demographic variables and blood glucose and were identified as diabetes participants or healthy participants according to the diagnostic criteria proposed by the American Diabetes Association (18). The severity of DR was graded by ophthalmologists from the Eye and ENT Hospital of Fudan University according to the Diabetic Retinopathy Early Treatment Study (19).

The characteristics of the included population are presented in **Table 1**. Among the healthy participants (mean [SD] age, 38 [14] years), 54% were female and among the diabetes participants (mean [SD] age, 70 [4] years), 57% were female.

**Table 1:** The characteristics of study population.

Characteristics	Unit	Healthy participants	Diabetes participants
		Mean (SD) or %	Mean (SD) or %
Age	Year	38 (14)	69 (4)
Gender	Female	54%	57%
	Male	46%	43%
Eyes	Left eyes	50%	49%
	Right eyes	50%	51%
Severity of diabetic retinopathy	No DR		34%
	Mild		48%
	Moderate		11%
	Severe		7%

The recruitment criteria of healthy participants were: (1) between 20 and 85 years of age, (2) long-term residence in Shanghai, (3) no adverse lifestyle habits, (4) no major illnesses, and (5) self-reported healthy.

The recruitment criteria of diabetes participants were: (1) between 20 and 85 years of age, and (2) having diabetes.

The image quality control with the following exclusion criteria: (1) signal intensity less than 6, (2) presence of motion artifacts, tomography, and dislocation, (3) image center deviation from the macula,(4) inaccurate vessel delamination, and (5) low image resolution.

### Measurement of Systemic Risk Factors

The systemic test included fasting blood test, urine test, blood pressure test, pulse test, vision test, and measurements of demographic variables. Indicators of fasting blood test were basophil count, basophil ratio, eosinophil count, eosinophil ratio, hematocrit, hemoglobin, lymphocyte count, lymphocyte ratio, mean corpuscular hemoglobin, mean cell hemoglobin concentration, monocyte count, mean platelet volume, plateletcrit, platelet distribution width, platelet larger cell ratio, platelet count, red cell distribution width coefficient of variation, neutrophilic granulocyte count, neutrophilic granulocyte ratio, creatinine, total cholesterol, glucose, high-density lipoprotein cholesterol, low-density lipoprotein cholesterol, total bilirubin, triglyceride, uric acid, and glycosylated hemoglobin. Indicators of blood pressure test were diastolic blood pressure and systolic blood pressure. The indicators of urine tests, pulse test, and vision test are urine specific gravity, pulse, and uncorrected visual acuity respectively. Demographic variables include age, sex, height, and weight. Height and weight were converted to body mass index (BMI) and uncorrected visual acuity was converted to logarithm of the minimum angle of resolution for analysis.

### Data Collection Based on OCTA and OCT

Swept-source OCT (DRI OCT Triton; Topcon Inc, Tokyo, Japan) was used in our study. The DRI OCT uses infrared light at a wavelength of 1,050 nm as the light source and achieves a high A-scan rate of 100 kHz, resulting in high signal penetration and fast imaging. An OCT scan of a 3 mm x 3 mm field of view takes approximately 4 s. The lateral resolution is 20 µm and the axial resolution is 8 µm. DRI OCT uses an en face visualization technology to reconstruct the blood flow signal in three dimensions. The embedded software IMAGEnet 6 can divide the retinal into superficial capillary plexus (SCP) and deep capillary plexus (DCP). SCP is segmented from the inner limiting membrane (offset 2.6 μm) to the junction (offset 15.6 μm) of the inner plexiform layer and inner nuclear layer. DCP is 15.6 μm down from the junction of the inner plexiform layer and the inner core layer to 70.2 μm.

Both eyes of every participant have been performed with OCT scans centered on the macula and optic disc, OCTA imaging centered on the macula with 3 mm x 3 mm area, and color fundus photograph. Among the 100 healthy participants from the Eye and ENT Hospital of Fudan University, 37 of them underwent four repeated OCTA imaging within 10 min to analyze the reliability of the segmentation method proposed in this article. All the OCTA images of the participants were submitted to the technicians for quality control.

### Segmentation and Quantification of the Retinal Vessels for OCTA Images

**Figure 1:**
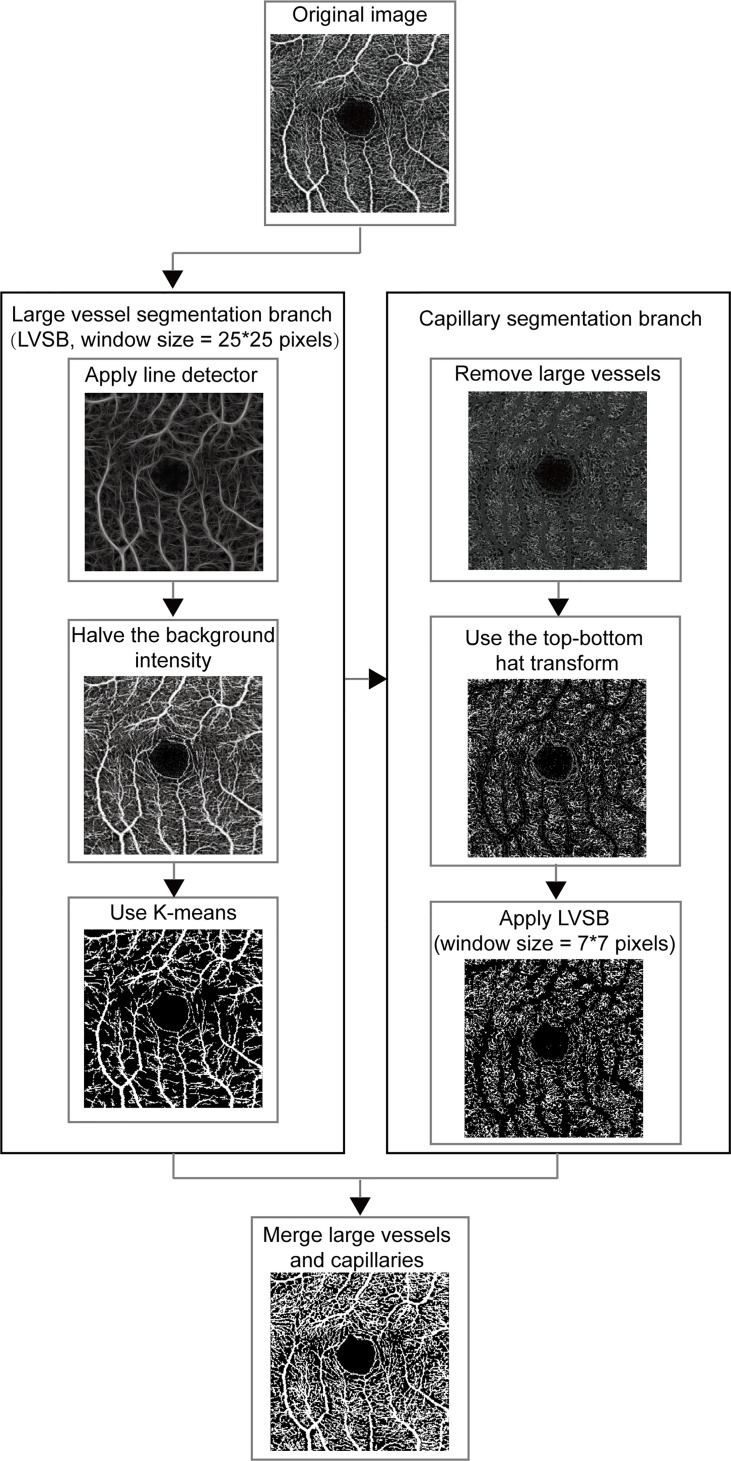
The framework for vessel segmentation method on OCTA image. LVSB, large vessel segmentation branch.

The OCTA images of SCP were exported in TIF format and automatically segmented. As depicted in **Figure 1**, MRVSM included a large vessel segmentation branch (LVSB), a capillary segmentation branch, and their fusion module. Considering that capillaries are difficult to distinguish from the background, we firstly segmented large vessels and then capillaries, where the segmentation methods are designed separately and their parameters adjusted accordingly. The benefit of this process is that it effectively reduces the segmentation error rate of capillaries and better suppresses noises.

Line detectors with different window sizes were used to distinguish between large vessels and capillaries, which was proposed in (20). The line detector computed the line strength of the target pixel which was defined as the largest average gray levels along the line going through the target pixel over the 12 directions (15° of the angular interval from 0°) in the set square window and was used to distinguish between vessel pixel and background pixel. The line detector was sensitive to different vessels according to the window size. In large vessel segmentation, the line detector with a window size of 25 x 25 pixels were first used to extract the structural features of large vessels. The linear combination of structural features extracted by the line detector and the intensity of vessels was binarized to roughly separate the large vessels and the backgrounds. The linear combination coefficients were 1/3 for structural features and 2/3 for intensity. The detected background intensity was halved to improve the contrast between the large vessels and the background. Vessel pixels and background ones were separated using K-means clustering according to their gray levels (21). The separation was simplified since the large vessels were enhanced.

In the capillary segmentation, we set the gray value of the detected large vessel pixels to 45 to eliminate the interference of large vessels. As the gray levels of the capillary pixels are close to the background pixels, pre-processing is required to enhance the capillaries. The top–bottom hat transform was first used to highlight capillary details and inhibit background which added the top hat transform effect and subtracted the bottom hat transform effect (22). Then we used the same process as LVSB, integrating line detector and k-means clustering to segment capillaries. In order to fit the capillary, the window size of the line detector in the capillary segmentation branch was smaller. It was empirically set to 7 x 7 pixels for capillary segmentation. In the fusion module, large vessels and capillaries, which are the foreground of the segmented images obtained from the two branches, were merged to generate the segmentation result.

### Quantification of OCTA Metrics of Retinal Vessel

The vessels on the OCTA images were segmented by MRVSM automatically, FAZ was detected by the method in (23), and a non-perfusion zone was detected by the method in (24). Approximately 24 OCTA metrics were quantified based on the extracted blood vessels, FAZ, and non-perfusion zone. Among the OCTA metrics, vessel skeleton fractal dimension was defined in (16). Vessel density, vessel skeleton density, vessel perimeter index, vessel diameter index, and vessel complexity index were defined in (17). Big vessel density, big vessel skeleton density, small vessel density, small vessel skeleton density, non-perfusion area, FAZ circularity index, and FAZ area were defined in (24). Vessel density in 1 mm circle and vessel density in 2 mm circle were defined in (25). FAZ perimeter, FAZ major axis diameter, FAZ minor axis diameter, and FAZ orientation were defined in (26). FAZ horizontal diameter and FAZ vertical diameter were defined in (27). Besides, we quantified three OCTA metrics, including vessel skeleton density in the 1 mm circle, vessel skeleton density in the 2 mm circle, and FAZ eccentricity. The above OCTA metrics were defined in the supplementary material (**Supplementary Material**).

Other OCTA metrics automatically calculated by the instrument included center vessel density in the superficial layer (CVDS), superior vessel density in the superficial layer, inferior vessel density in the superficial layer, nasal vessel density in the superficial layer, temporal vessel density in the superficial layer, center vessel density in the deep layer, superior vessel density in the deep layer (SVDD), inferior vessel density in the deep layer (IVDD), nasal vessel density in the deep layer, and temporal vessel density in the deep layer, where they were within the circle with a 2.5-mm diameter centered on the center of foveal avascular zone, optic disc area, optic disc volume, optic cup area, optic cup volume, optic rim area, optic rim volume, the area ratio of cup and disc, the square root of the area ratio of cup and disc, the vertical diameter ratio of cup and disc, disc vertical diameter, disc horizontal diameter, mean thickness of macular in the retina layer, mean thickness of macular in the retinal nerve fiber layer (MTMRNFL), mean thickness of macular in the ganglion cell layer with inner plexiform layer (MTMGCL+), mean thickness of macular in the ganglion cell layer with inner plexiform layer and nerve fiber layer, mean thickness of macular in the choroidal layer, mean thickness of optic disc in the retina layer, mean thickness of optic disc in the retinal nerve fiber layer (MTODRNFL), mean thickness of optic disc in the ganglion cell layer with inner plexiform layer, mean thickness of optic disc in the ganglion cell layer with inner plexiform layer and nerve fiber layer (MTODGCL++), and mean thickness of optic disc in the choroidal layer (MTODCL).

### Evaluation and Statistical Analysis

We used each eye with the inclusion criteria as a subject for analysis and calculated its OCTA metrics. All statistical analyses were performed in R. To ensure the quality of the data, histograms, box plots and scatter plots were made for quality control and checked by visual inspection. The data were preprocessed to replace the null values with the mean of other samples in the same group. To assess the reliability and reproducibility of the OCTA metrics based on MRVSM, Within-subject standard deviation (Sw) (28), coefficient of variation (CoV, 100 × Sw/overall mean) (28), and intraclass correlation coefficient (ICC) (29) were calculated. To assess the interocular correlation of the OCTA metrics, t-test was used to analyze whether the OCTA metrics were statistically different between eyes. Then, Pearson correlation coefficient, ICC, and Bland–Altman plots (30) were used to evaluate the interocular correlation. Univariate linear regression models with OCTA metrics as the dependent variables and systemic risk factors as independent variables were used to assess the effect of systemic risk factors on OCTA metrics. The significances (P-values) of univariate linear regression were corrected for FDR (31). For each diabetes case, we choose one matched control from the healthy group with replacement to make the identified systemic risk factors of the two cases as closely as possible. The distance of the two individuals was measured by Euclidean distance. Then, Wilcox test and FDR correction were used to test for differences in OCTA metrics between diabetic and healthy controls. Stepwise regression with Akaike information criterion (AIC) as objective function which repeatedly added most important independent variable or removed variables that are not important and highly correlated with other variables was used to build the model. Here, we used stepwise regression to determine the important OCTA metrics which were the independent variables of the logistic regression model to classify the DR participants and diabetes participants free of DR. Similarly, the OCTA metrics related to severe DR were found by stepwise regression which were the independent variables of the logistic regression model to classify severe DR participants and non-severe DR participants. The prediction powers of the models were measured by averaging the receiver operating characteristic curves (ROC), the area under the curve (32) (AUC) values, and the F_1_ scores (33) under the 5-fold cross validation.

## Results

### Assessment of MRVSM

**Figure 2:**
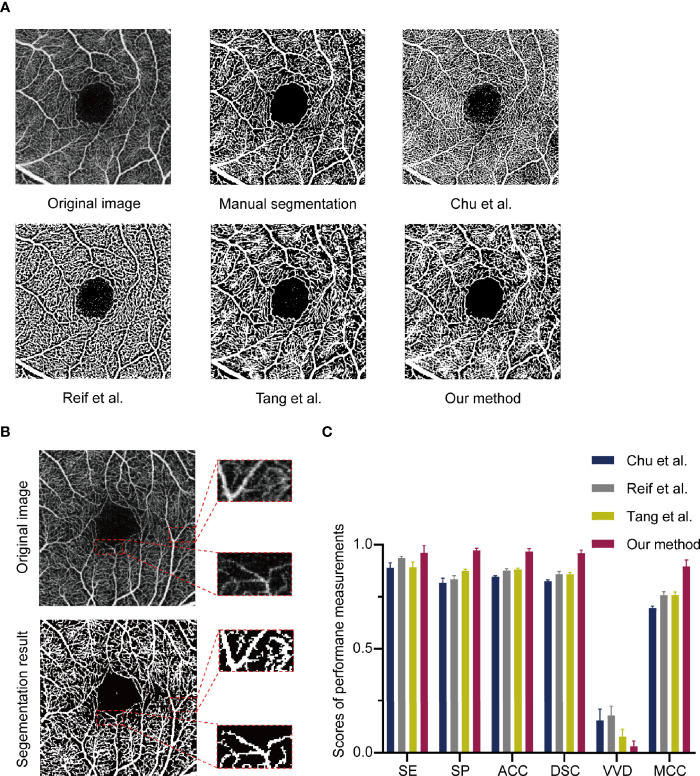
The assessment of the multi-branch retinal vessel segmentation method (MRVSM). **(A)** Comparison of results of segmentation methods. **(B)** Segmentation results of MRVSM in the low contrast region. **(C)** The scores of sensitivity (SE), specificity (SP), accuracy (ACC), absolute vessel volume difference (VVD), dice similarity coefficient (DSC), and Matthews correlation coefficient (MCC) of the segmentation methods. Data were presented as the mean ± SD.

Manually annotated were 11 OCTA images as ground truth and the performance of MRVSM was evaluated. The evaluation metrics included sensitivity, specificity, accuracy, the absolute vessel volume difference (34), the dice similarity coefficient (35), and the Matthews correlation coefficient (36). Results show that MRVSM is close to the manual segmentation and outperforms state-of-the-art methods such as Reif et al. (16), Chu et al. (17), and Tang et al. (14) (**Figure 2A**). Apart from that, MRVSM can extract the blood vessels better in the low contrast area (**Figure 2B**). Compared with the state-of-the-art ones, MRVSM has the highest accuracy rate and the lowest error rate, which illustrates the advanced nature of MRVSM (**Figure 2C**). The performance evaluation data of the segmentation methods are shown in the supplementary material (**Supplementary Table 1**).

### Reliability and Repeatability Analysis of OCTA Metrics Based on MRVSM

**Figure 3:**
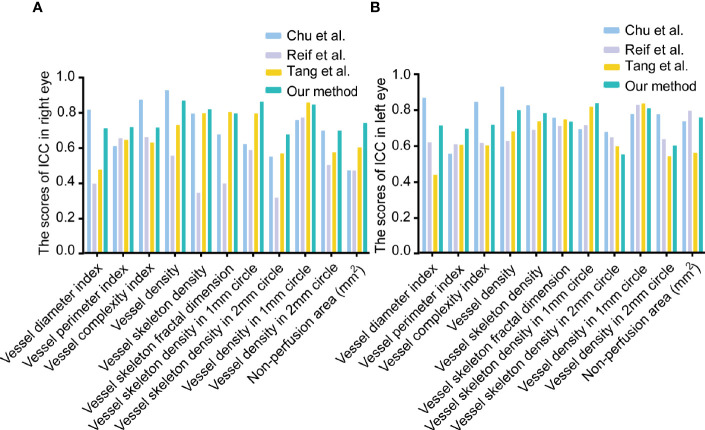
The scores of intraclass correlation coefficient (ICC) of OCTA quantitative metrics. **(A)** The scores of ICC in the right eyes. **(B)** The scores of ICC in the left eyes.

In the reliability analysis, the OCTA metrics based on MRVSM included big vessel density, small vessel density, big vessel skeleton density, small vessel skeleton density, vessel density, vessel skeleton density, vessel skeleton fractal dimension, vessel perimeter index, vessel diameter index, non-perfusion area, vessel complexity index, vessel skeleton density in the 1 mm circle, vessel density in 1 mm circle, vessel skeleton density in the 2 mm circle, and vessel density in 2 mm circle. Since Chu et al. (17), Reif et al. (16), and Tang et al. (14) cannot separate large and small blood vessels, we did not analyze the reliability and repeatability of big vessel skeleton density, small vessel skeleton density, big vessel skeleton density, and small vessel skeleton density. The eyes of 37 healthy participants from the Eye and ENT Hospital of Fudan University underwent four repeated OCTA imaging within 10 min. Sw and CoV were used to evaluate the repeatability of OCTA metrics, where low Sw and low CoV mean high repeatability. ICC was used to evaluate the reliability of OCTA metrics, where high ICC means high reliability. For the repeatability analysis of OCTA metrics, MRVSM had the lowest scores of Sw in vessel skeleton density and vessel skeleton density in the 1 mm circle of the right eyes (**Supplementary Table 2**). **Figure 3** shows the scores of ICC of the OCTA metrics. For the vessel perimeter index and the vessel skeleton density in the 1 mm circle of the left eyes, and vessel perimeter index, vessel skeleton density, non-perfusion area, vessel skeleton density in the 1 mm circle, vessel skeleton density in the 2 mm circle, and vessel density in 2 mm circle of the right eyes, MRVSM has the highest scores of ICC (**Figure 3**). In addition, the ICC ranking of other OCTA quantitative metrics based on MRVSM is not at the bottom (**Figure 3**).

### The Relationship Between Systemic Risk Factors and OCTA Metrics

**Figure 4:**
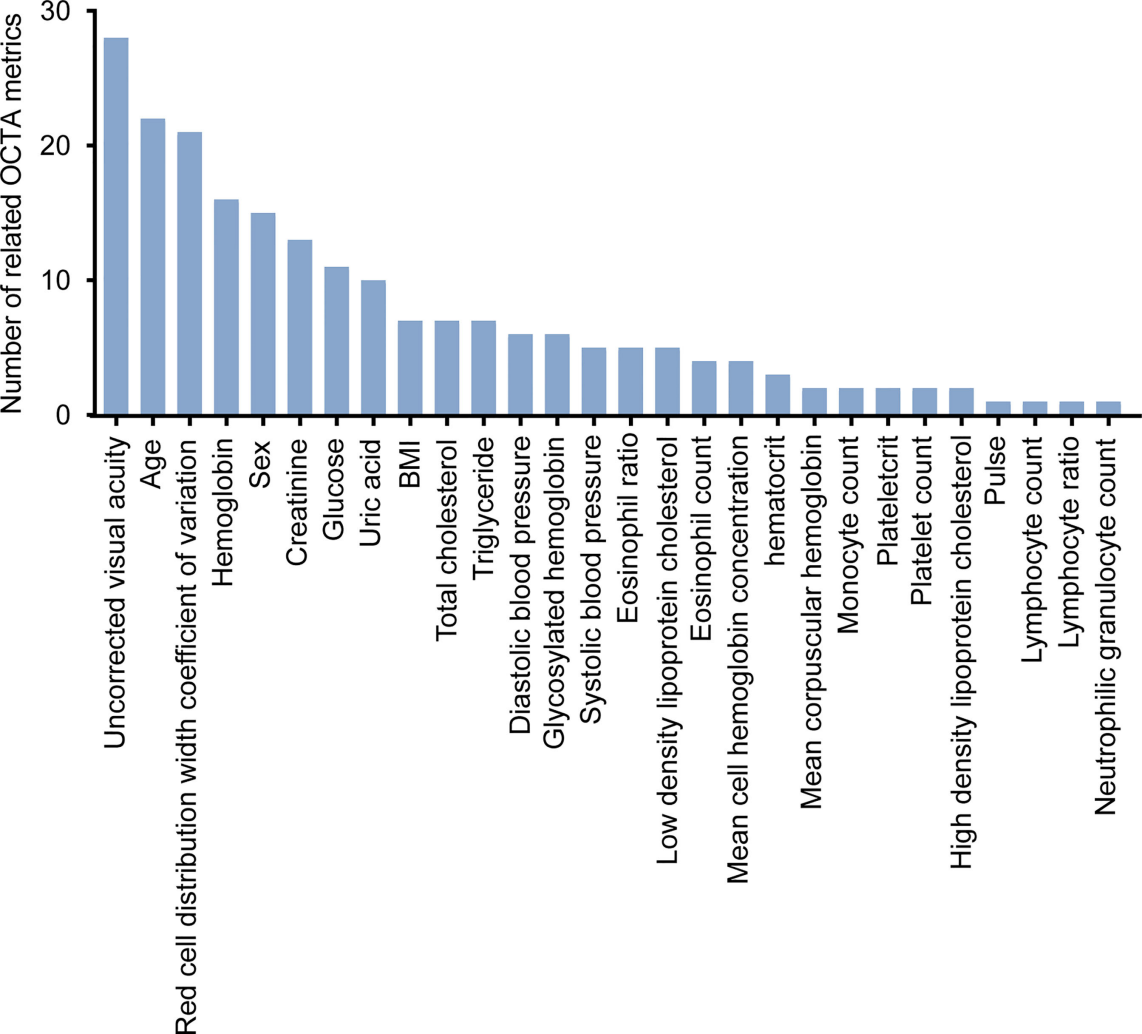
The systemic risk factors associated with OCTA metrics. FDR-corrected P-values were calculated by Wilcox test.

Approximately 1,077 eyes of 550 healthy participants who underwent the measurement of systemic risk factors were used for the analysis of the relationship between systemic risk factors and all OCTA metrics. These participants have the following characteristics: females account for 55%, and age ranges from 20 to 79 years (mean [SD] age, 36 [14] years). Systemic risk factors associated with OCTA metrics were identified by the univariate linear regression model (F test). The results of the above correlation analysis were subjected to the Kolmogorov–Smirnov test to confirm the symmetry of the residuals of the univariate linear regression model and the FDR correction to adjust the p-values of the univariate linear regression model. Found to be associated with OCTA metrics were 28 systemic risk factors (**Figure 4**). The number of systemic risk factors which were strongly associated with OCTA metrics was calculated as 8 by the method described in (37). The eight systemic risk factors are uncorrected visual acuity, age, red cell distribution width coefficient of variation, hemoglobin, sex, creatinine, glucose, and uric acid. The results of the univariate linear regression model, Kolmogorov–Smirnov test, and FDR correction used to analyze the relationship between OCTA metrics and systemic risk factors are shown in **Supplementary Table 3**.

### Analysis of OCTA Metrics Related to Diabetes

**Figure 5:**
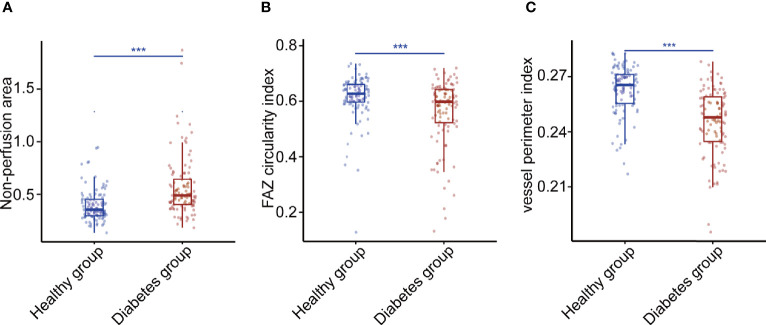
Three OCTA metrics closely related to diabetes. **(A)** The Wilcox result of non-perfusion area. **(B)** The Wilcox result of foveal avascular zone circularity index. **(C)** The Wilcox result of vessel perimeter index. P-values were corrected by FDR. *p < 0.05, **p < 0.01, and ***p < 0.001. FAZ, foveal avascular zone.

For each diabetes case, we choose one matched control from the healthy group with replacement to make the eight identified systemic risk factors of the two cases as closely as possible. Approximately 115 eyes of 68 diabetes participants (56% women; mean [SD] age, 70 [4] years) and 115 eyes of 81 healthy people (53% women; mean [SD] age, 36 [13] years) were used to analyze the relationship between OCTA metrics and diabetes. Approximately 34 OCTA metrics were found to be significantly different between the diabetes case and healthy case by Wilcox test and FDR correction (**Supplementary Table 4**). **Figure 5** shows the Wilcox results of non-perfusion area, FAZ circularity index, and vessel perimeter index which had the close relationships with diabetes (FDR-corrected P-values <0.001).

### Analysis of OCTA Metrics Related to the Severity of DR

We further analyzed whether the above 34 OCTA metrics were related to DR and severe DR. This analysis was based on the 362 eyes of the 221 diabetes participants. Of the 362 eyes with diabetes, 172 (48%) eyes were mild non-proliferative diabetic retinopathy (NPDR), 41 (11%) eyes were moderate NPDR, and 27 (7%) eyes were severe NPDR.

**Figure 6:**
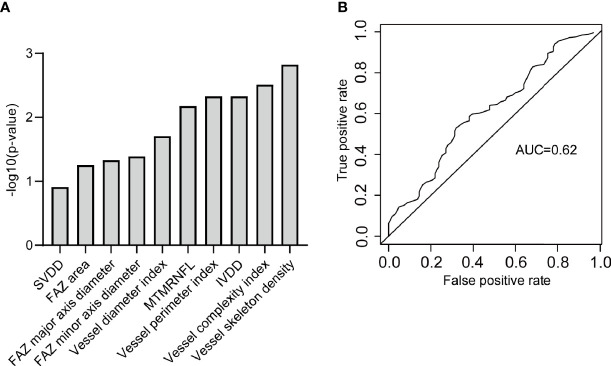
The OCTA metrics related to DR. **(A)** The OCTA metrics related to DR and identified by stepwise regression. **(B)** The ROC curve of the logistic regression model to classify DR participants and diabetic free of DR participants. SVDD, superior vessel density in the deep layer; FAZ, foveal avascular zone; MTMRNFL, mean thickness of macular in the retinal nerve fiber layer; IVDD, inferior vessel density in the deep layer.

**Figure 7:**
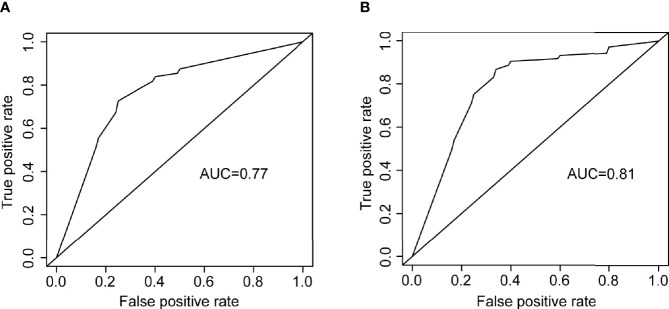
The ROC curves of two logistic regression models for distinguishing DR participants from diabetes participants free of DR. **(A)** the logistic regression using systemic risk factors as independent variables. **(B)** the logistic regression using OCTA metrics as independent variables.

SVDD, FAZ area, FAZ major axis diameter, FAZ minor axis diameter, vessel diameter index, MTMRNFL, vessel perimeter index, IVDD, vessel complexity index, and vessel skeleton density were found to be related to DR by stepwise regression (**Figure 6A**). The logistic regression model with the above metrics as independent variables further proved that they were closely related to DR (**Figure 6B**). To prove that the above OCTA metrics did correlate with DR, we first made a logistic regression using the systemic risk factors as independent variables to distinguish DR participants with diabetes participants free of DR as a baseline. A stepwise regression scheme was used to determine which systemic risk factors were included in the model. The model got an average AUC of 0.77 and an F_1_ score of 0.73 under 5-fold cross validation (**Figure 7A**). Then we added the above OCTA metrics to the baseline model. Another round of stepwise regression was applied to rebuild the model. The performances of the models were improved (with AUC improved into 0.81 and F_1_ score improved into 0.87, **Figure 7B**). These results that OCTA metrics selected in this section did correlate to the DR, even after the information of them encoded in systemic metrics deduced.

**Figure 8:**
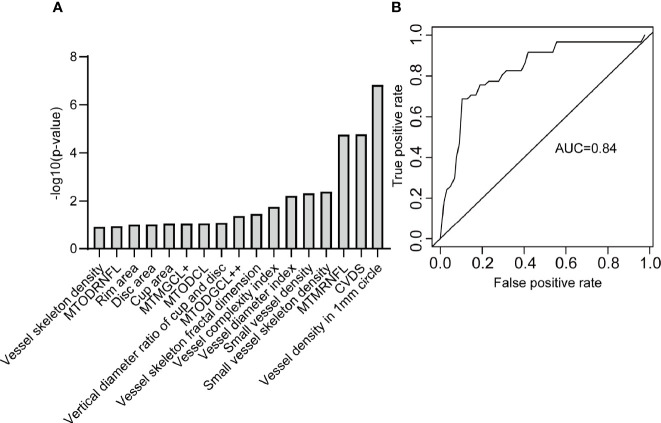
The OCTA metrics related to severe DR. **(A)** The OCTA metrics related to severe DR and identified by stepwise regression. **(B)** The ROC curve of the logistic regression model to distinguish severe DR participants from non-severe DR participants. MTODRNFL, mean thickness of optic disc in the retinal nerve fiber layer; MTMGCL+, mean thickness of macular in the ganglion cell layer with inner plexiform layer; MTODCL, mean thickness of optic disc in the choroidal layer; MTODGCL++, mean thickness of optic disc in the ganglion cell layer with inner plexiform layer and nerve fiber layer; MTMRNFL, mean thickness of macular in the retinal nerve fiber layer; CVDS, center vessel density in the superficial layer.

**Figure 8** shows the ROC curve of the OCTA metrics associated with severe DR. There was a strong correlation between severe DR and vessel skeleton density, rim area, disc area, cup area, MTODRNFL, MTMGCL+, MTODCL, vertical diameter ratio of cup and disc, MTODGCL++, vessel skeleton fractal dimension, vessel complexity index, vessel diameter index, small vessel density, small vessel skeleton density, MTMRNFL, CVDS, and vessel density in 1 mm circle (**Figure 8A**). The logistic regression model constructed from these OCTA metrics can effectively distinguish severe DR participants from the non-severe DR participants with an accuracy of 0.80 and an AUC of 0.84 (**Figure 8B**).

## Discussion

In this study, we developed a multi-stage vessel segmentation method (MRVSM) which achieved effective segmentation on low-quality capillary and was comparable to manual segmentation. The reliability of OCTA metrics based on MRVSM is not inferior to that of OCTA metrics based on Reif et al. (16), Chu et al. (17), and Tang et al. (14). This provides a basis for further analysis of the relationship between OCTA metrics and diabetes and DR. We found eight systemic risk factors that are strongly correlated with OCTA metrics. After controlling for the eight systemic risk factors, we found 34 OCTA metrics related to diabetes, such as non-perfusion area, FAZ circularity index, and vessel perimeter index. Among the 34 OCTA metrics, 10 OCTA metrics (such as FAZ area, vessel diameter index, mean thickness of retinal nerve fiber layer centered in the macular) were found to be related to DR which can distinguish DR participants from diabetes participants free of DR even after the prediction power of systemic risk factors discounted. It is noteworthy that 17 OCTA metrics (such as vessel complexity index, vessel diameter index, and mean thickness of retinal nerve fiber layer centered in the macular) were found to be related to severe DR. The model constructed from the 17 OCTA metrics can effectively distinguish the severe DR from non-severe DR. This study provides evidence to support the potential value of OCTA metrics in assessing and monitoring DR progression.

Although previous works had studied the OCTA metrics and the severity of DR, our study has precise automated blood vessel segmentation software, comprehensive quantification of OCTA metrics, a large number of samples, comprehensive physical examination data, and strict image quality control standards. Since the proposed segmentation method is not inferior to existing segmentation algorithms, it has better application prospects and provides a new idea in vessel segmentation fields. The OCTA metrics found related to DR can help clinicians to screen DR patients, thus facilitating early detection of DR patients and delaying the development of DR. It is noteworthy that the OCTA metrics found related to severe DR facilitate timely detection and therapeutic intervention for patients with severe NPDR, which can prevent blindness. The above conclusions are beneficial for people to better understand the changes in the retinal fundus caused by DR. The significant difference of OCTA metrics in different severity of DR is conducive to monitoring and management of DR, and has the potential to become a biomarker of DR.

There are still some shortcomings that need improvement. The OCTA image size of the macula in this paper is relatively small (3 mm x 3 mm), leading to the inability to obtain some vessel quantitative metrics. Large size means low resolution, so a balance between size and resolution is needed. In the follow-up study, we will increase the subject’s population diversity and extend MRVSM to perform on large-scale images. Second, we only included high-resolution images, which may introduce bias. Third, the ratio of patients with moderate DR, severe DR is relatively small. We will recruit more participants with larger diversity in the follow-up studies.

## Data Availability Statement

The original contributions presented in the study are included in the article/**Supplementary Material**. Further inquiries can be directed to the corresponding authors.

## Ethics Statement

The studies involving human participants were reviewed and approved by the Ethics Committee of Eye and ENT Hospital of Fudan University and the Ethics Committee of School of Life Sciences of Fudan University. The participants provided their written informed consent to participate in this study.

## Author Contributions

BingX, ZY, and ZC conceived the project and designed the study. JC and SZ recruited volunteers from the Eye and ENT Hospital of Fudan University to collect their data. JC graded the severity of DR on OCTA images. SS and XL collected volunteers’ OCT and OCTA data at the Human Phenome Institute of Fudan University. BinX proposed the automatic segmentation method and analyzed all the data, and wrote the manuscript. BingX, ZY, ZC, BinX, JC, SZ, SS, and XL revised the manuscript. All authors contributed to the article and approved the submitted version.

## Funding

This study was supported by funds from the Shanghai Municipal Science and Technology Major Project (Grant No. 2017SHZDZX01 and No.2018SHZDZX01) and ZJLab, the National Key R&D Program of China Project (2017YFA0103900), the National Natural Science Foundation of China (31571083, 31970931), the Program for Professor of Special Appointment (Eastern Scholar of Shanghai, TP2014008), and the Shanghai Rising-Star Program (14QA1400800). This study received funding from State Key Laboratory of Translational Medicine and Innovative Drug Development, Jiangsu Simcere Diagnostics Co., Ltd. and Nanjing Simcere Medical Laboratory Science Co., Ltd. The funder was not involved in the study design, collection, analysis, interpretation of data, the writing of this article or the decision to submit it for publication. All authors declare no other competing interests.

## Conflict of Interest

Author BingX was employed by company Jiangsu Simcere Diagnostics Co., Ltd., and company Nanjing Simcere Medical Laboratory Science Co., Ltd.

The remaining authors declare that the research was conducted in the absence of any commercial or financial relationships that could be construed as a potential conflict of interest.

## Publisher’s Note

All claims expressed in this article are solely those of the authors and do not necessarily represent those of their affiliated organizations, or those of the publisher, the editors and the reviewers. Any product that may be evaluated in this article, or claim that may be made by its manufacturer, is not guaranteed or endorsed by the publisher.
