# Supplementary material for: Association Between the Severity of Diabetic Retinopathy and Optical Coherence Tomography Angiography Metrics
Source: Front Endocrinol (Lausanne). 2021 Dec 10;12:777552. doi: 10.3389/fendo.2021.777552 (PMC8702651; doi:10.3389/fendo.2021.777552)
Supplement: Supplementary file 1 [file DataSheet_1.docx]

**Supplementary Material**

Vessel density (VAD) is the area ratio of vessels and total image, defined as：

$$\boldsymbol{VAD}\mathbf{=}\frac{\sum_{\boldsymbol{i=1,j=1}}^{\boldsymbol{n}} \boldsymbol{V(i,j)}}{\boldsymbol{N}}$$

Where $V(i,j)$ represents the pixels of vessels and $N$ represents the number of all pixels in the image.

Vessel skeleton density (VSD) is the ratio of the length of vessels and total image area, defined as:

$$\boldsymbol{VSD}\mathbf{=}\frac{\sum_{\boldsymbol{i=1,j=1}}^{\boldsymbol{n}} \boldsymbol{S(i,j)}}{\boldsymbol{N}}$$

Where $S(i,j)$ represents the pixels of vessel skeleton and $N$ represents the number of all pixels in the image.

Vessel skeleton fractal dimension (FD) is calculated by box counting, defined as:

$$\boldsymbol{FD=-}\frac{\log\boldsymbol{N}_{\boldsymbol{r}}\boldsymbol{}}{\log\boldsymbol{r}}$$

Where $N_{r}$ is number of boxes which is used to cover the vessel skeleton and $r$ is the box length.

Vessel perimeter index (VPI) is the ratio of the perimeter of vessels and total image area, defined as:

$$\boldsymbol{VPI}\mathbf{=}\frac{\sum_{\boldsymbol{i=1,j=1}}^{\boldsymbol{n}} \boldsymbol{P(i,j)}}{\boldsymbol{N}}$$

Where $P(i,j)$ represents the pixels of the outline of vessels and $N$ represents the number of all pixels in the image.

Vessel diameter index (VDI) is the mean vessel diameter, which is defined as:

$$\boldsymbol{VDI}\mathbf{=}\boldsymbol{L}_{\boldsymbol{pixel}}\boldsymbol{\times}\frac{\sum_{\boldsymbol{i=1,j=1}}^{\boldsymbol{m}} \boldsymbol{V(i,j)}}{\sum_{\boldsymbol{i=1,j=1}}^{\boldsymbol{n}} \boldsymbol{S(i,j)}}$$

Where $L_{pixel}$ represents the length of one pixel, $V(i,j)$ represents the pixels of vessels, and $S(i,j)$ represents the pixels of vessel skeleton.

Vessel complexity index (VCI) is defined as:

$$\boldsymbol{VCI}\mathbf{=}\frac{{\boldsymbol{(}\sum_{\boldsymbol{i=1,j=1}}^{\boldsymbol{m}} \boldsymbol{P(i,j))}}^{\boldsymbol{2}}}{\boldsymbol{4}\boldsymbol{\pi}\sum_{\boldsymbol{i=1,j=1}}^{\boldsymbol{n}} \boldsymbol{V(i,j)}}$$

Where $P(i,j)$ represents the pixels of the outline of vessels, $V(i,j)$ represents the pixels of vessels.

Big vessel density (BVD) is defined as:

$$\boldsymbol{BVD}\mathbf{=}\frac{\sum_{\boldsymbol{i=1,j=1}}^{\boldsymbol{n}} \boldsymbol{V}_{\boldsymbol{big}}\boldsymbol{(i,j)}}{\boldsymbol{N}}$$

Where $V_{big}(i,j)$ represents the pixels of big vessels and $N$ represents the number of all pixels in the image.

Big vessel skeleton density (BSD) is defined as:

$$\boldsymbol{BSD}\mathbf{=}\frac{\sum_{\boldsymbol{i=1,j=1}}^{\boldsymbol{n}} \boldsymbol{S}_{\boldsymbol{big}}\boldsymbol{(i,j)}}{\boldsymbol{N}}$$

Where $S_{big}(i,j)$ represents the pixels of big vessel skeleton and $N$ represents the number of all pixels in the image.

Small vessel density (SVD) is defined as:

$$\boldsymbol{SVD}\mathbf{=}\frac{\sum_{\boldsymbol{i=1,j=1}}^{\boldsymbol{n}} \boldsymbol{V}_{\boldsymbol{small}}\boldsymbol{(i,j)}}{\boldsymbol{N}}$$

Where $V_{small}(i,j)$ represents the pixels of small vessels and $N$ represents the number of all pixels in the image.

Small vessel skeleton density (SSD) is defined as:

$$\boldsymbol{SSD}\mathbf{=}\frac{\sum_{\boldsymbol{i=1,j=1}}^{\boldsymbol{n}} \boldsymbol{S}_{\boldsymbol{small}}\boldsymbol{(i,j)}}{\boldsymbol{N}}$$

Where $S_{small}(i,j)$ represents the pixels of small vessel skeleton and $N$ represents the number of all pixels in the image.

Vessel density in 1mm circle (VD1) is defined as:

$$\boldsymbol{VD}\boldsymbol{1}\mathbf{=}\frac{\sum_{\boldsymbol{i=1,j=1}}^{\boldsymbol{n}} \boldsymbol{V}_{\boldsymbol{1}\boldsymbol{mm}}\boldsymbol{(i,j)}}{\boldsymbol{N}}$$

Where $V_{1mm}(i,j)$ represents the vessel pixels in the macular within a circle of 1 mm diameter and $N$ represents the number of all pixels in the image.

Vessel density in 1mm circle (VD2) is defined as:

$$\boldsymbol{VD}\boldsymbol{2}\mathbf{=}\frac{\sum_{\boldsymbol{i=1,j=1}}^{\boldsymbol{n}} \boldsymbol{V}_{\boldsymbol{2}\boldsymbol{mm}}\boldsymbol{(i,j)}}{\boldsymbol{N}}$$

Where $V_{2mm}(i,j)$ represents the vessel pixels in the macular within a circle of 2 mm diameter and $N$ represents the number of all pixels in the image.

The unit of non-perfusion area (NP) is square millimeter, which is defined as:

$$\boldsymbol{NP}\mathbf{=}\boldsymbol{A}_{\boldsymbol{image}}\boldsymbol{\times}\frac{\sum_{\boldsymbol{i=1,j=1}}^{\boldsymbol{n}} \boldsymbol{NoP(i,j)}}{\boldsymbol{N}}$$

Where $A_{image}$ represents the area of the image, $NoP(i,j)$ represents the pixels of non-perfusion, and $N$ represents the number of all pixels in the image.

Vessel skeleton density in 1mm circle (SD1) is defined as:

$$\boldsymbol{SD}\boldsymbol{1}\mathbf{=}\frac{\sum_{\boldsymbol{i=1,j=1}}^{\boldsymbol{n}} \boldsymbol{S}_{\boldsymbol{1}\boldsymbol{mm}}\boldsymbol{(i,j)}}{\boldsymbol{N}}$$

Where $S_{1mm}(i,j)$ represents the vessel skeleton pixels in the macular within a circle of 1 mm diameter and $N$ represents the number of all pixels in the image.

vessel skeleton density in 2mm circle (SD2) is defined as:

$$\boldsymbol{SD}\boldsymbol{2}\mathbf{=}\frac{\sum_{\boldsymbol{i=1,j=1}}^{\boldsymbol{n}} \boldsymbol{S}_{\boldsymbol{2}\boldsymbol{mm}}\boldsymbol{(i,j)}}{\boldsymbol{N}}$$

Where $S_{2mm}(i,j)$ represents the vessel skeleton pixels in the macular within a circle of 2 mm diameter and $N$ represents the number of all pixels in the image.

Foveal avascular zone (FAZ) area is defined as:

$$\boldsymbol{FAZ area}\mathbf{=}\boldsymbol{A}_{\boldsymbol{image}}\boldsymbol{\times}\frac{\sum_{\boldsymbol{i=1,j=1}}^{\boldsymbol{n}} \boldsymbol{F(i,j)}}{\boldsymbol{N}}$$

Where $A_{image}$ represents the area of the image, $F(i,j)$ represents the pixels in foveal avascular zone, and $N$ represents the number of all pixels in the image.

FAZ perimeter is defined as:

$$\boldsymbol{FAZ}\mathbf{perimeter=}\boldsymbol{L}_{\boldsymbol{pixel}}\boldsymbol{\times}\sum_{\boldsymbol{i=1,j=1}}^{\boldsymbol{n}} \boldsymbol{P}_{\boldsymbol{FAZ}}\boldsymbol{(i,j)}$$

Where $L_{pixel}$ represents the length of one pixel and $P_{FAZ}(i,j)$ represents the pixels of the outline of FAZ.

Foveal avascular zone circularity index (FAZCI) is defined as:

$$\boldsymbol{FAZCI}\mathbf{=}\frac{\boldsymbol{4\times\pi\times area}}{\boldsymbol{perimeter}^{\boldsymbol{2}}}$$

Where $area$ is the area of FAZ, $perimeter$ is the perimeter of FAZ.

FAZ major axis diameter, FAZ minor axis diameter, FAZ orientation, FAZ eccentricity are the long axis length, short axis length, eccentricity and the angle between the long axis and the horizontal line of the ellipse with the same second-order central moment as FAZ, respectively. FAZ horizontal diameter and FAZ vertical diameter are the length and width of the circumscribed rectangle of FAZ, respectively

**Supplementary Table 1.** The performance of the published methods and the proposed method in this article.

The performance of the proposed method

| **Image No.** | **SE** | **SP** | **ACC** | **DSC** | **VVD** | **MCC** |
| --- | --- | --- | --- | --- | --- | --- |
| 1 | 0.9881 | 0.9792 | 0.9827 | 0.9786 | 0.0193 | 0.934 |
| 2 | 0.9588 | 0.9783 | 0.9707 | 0.9624 | 0.0075 | 0.9063 |
| 3 | 0.9594 | 0.9661 | 0.9634 | 0.9547 | 0.0097 | 0.8768 |
| 4 | 0.992 | 0.9659 | 0.9764 | 0.9712 | 0.0429 | 0.9032 |
| 5 | 0.9878 | 0.9758 | 0.9805 | 0.9752 | 0.0259 | 0.9226 |
| 6 | 0.9836 | 0.9745 | 0.978 | 0.9719 | 0.0241 | 0.9151 |
| 7 | 0.9401 | 0.9902 | 0.9697 | 0.962 | 0.0456 | 0.9239 |
| 8 | 0.9216 | 0.9638 | 0.9457 | 0.9356 | 0.03 | 0.845 |
| 9 | 0.8803 | 0.9786 | 0.9364 | 0.9224 | 0.0912 | 0.8459 |
| 10 | 0.971 | 0.9777 | 0.975 | 0.9695 | 0.0031 | 0.9175 |
| 11 | 0.9888 | 0.9487 | 0.9658 | 0.961 | 0.0579 | 0.8671 |

SE, sensitivity; SP, specificity; ACC, accuracy; DSC, Dice similarity coefficient; VVD, absolute vessel volume difference; MCC, Matthews correlation coefficient.

The performance of Chu et al.

| **Image No.** | **SE** | **SP** | **ACC** | **DSC** | **VVD** | **MCC** |
| --- | --- | --- | --- | --- | --- | --- |
| 1 | 0.8736 | 0.8274 | 0.8459 | 0.8191 | 0.1331 | 0.6898 |
| 2 | 0.9082 | 0.8155 | 0.8518 | 0.8277 | 0.1948 | 0.7080 |
| 3 | 0.9102 | 0.8166 | 0.8543 | 0.8341 | 0.1826 | 0.7137 |
| 4 | 0.8977 | 0.8112 | 0.8459 | 0.8240 | 0.1789 | 0.6960 |
| 5 | 0.8914 | 0.8250 | 0.8508 | 0.8228 | 0.1667 | 0.7015 |
| 6 | 0.8892 | 0.8135 | 0.8427 | 0.8137 | 0.1856 | 0.6867 |
| 7 | 0.8735 | 0.8263 | 0.8456 | 0.8218 | 0.1257 | 0.6901 |
| 8 | 0.8658 | 0.8302 | 0.8454 | 0.8273 | 0.0931 | 0.6901 |
| 9 | 0.8571 | 0.8513 | 0.8538 | 0.8343 | 0.0546 | 0.7045 |
| 10 | 0.8819 | 0.8212 | 0.8461 | 0.8243 | 0.1398 | 0.6930 |
| 11 | 0.9403 | 0.7628 | 0.8386 | 0.8326 | 0.2586 | 0.6974 |

The performance of Reif et al.

| **Image No.** | **SE** | **SP** | **ACC** | **DSC** | **VVD** | **MCC** |
| --- | --- | --- | --- | --- | --- | --- |
| 1 | 0.9415 | 0.8292 | 0.8741 | 0.8567 | 0.1982 | 0.7557 |
| 2 | 0.9397 | 0.8254 | 0.8702 | 0.8501 | 0.2110 | 0.7479 |
| 3 | 0.9414 | 0.8310 | 0.8754 | 0.8588 | 0.1923 | 0.7582 |
| 4 | 0.9402 | 0.8307 | 0.8747 | 0.8577 | 0.1924 | 0.7566 |
| 5 | 0.9449 | 0.8226 | 0.8702 | 0.8498 | 0.2239 | 0.7491 |
| 6 | 0.9431 | 0.8049 | 0.8583 | 0.8371 | 0.2533 | 0.7287 |
| 7 | 0.9401 | 0.8378 | 0.8795 | 0.8642 | 0.1757 | 0.7652 |
| 8 | 0.9266 | 0.8446 | 0.8797 | 0.8682 | 0.1345 | 0.7635 |
| 9 | 0.9232 | 0.8371 | 0.8741 | 0.8629 | 0.1396 | 0.7528 |
| 10 | 0.9411 | 0.8375 | 0.8799 | 0.8652 | 0.1754 | 0.7663 |
| 11 | 0.9276 | 0.8777 | 0.8990 | 0.8869 | 0.0917 | 0.7985 |

The performance of Tang et al.

| **Image No.** | **SE** | **SP** | **ACC** | **DSC** | **VVD** | **MCC** |
| --- | --- | --- | --- | --- | --- | --- |
| 1 | 0.9083 | 0.8729 | 0.8870 | 0.8653 | 0.0994 | 0.7710 |
| 2 | 0.8805 | 0.8801 | 0.8803 | 0.8521 | 0.0667 | 0.7529 |
| 3 | 0.9004 | 0.8670 | 0.8804 | 0.8584 | 0.0978 | 0.7578 |
| 4 | 0.9119 | 0.8616 | 0.8818 | 0.8611 | 0.1181 | 0.7623 |
| 5 | 0.9117 | 0.8707 | 0.8867 | 0.8621 | 0.1151 | 0.7696 |
| 6 | 0.9001 | 0.8745 | 0.8844 | 0.8574 | 0.0996 | 0.7630 |
| 7 | 0.8839 | 0.8864 | 0.8854 | 0.8628 | 0.0490 | 0.7651 |
| 8 | 0.8646 | 0.8786 | 0.8726 | 0.8530 | 0.0270 | 0.7408 |
| 9 | 0.8398 | 0.8899 | 0.8684 | 0.8457 | 0.0140 | 0.7311 |
| 10 | 0.9025 | 0.8674 | 0.8817 | 0.8621 | 0.0938 | 0.7613 |
| 11 | 0.9186 | 0.8713 | 0.8915 | 0.8785 | 0.0914 | 0.7832 |

**Supplementary Table 2.** Reliability and repeatability analysis of OCTA metrics.

Reliability and repeatability analysis of OCTA metrics in the right eyes.

|  | **Sw** | **CoV** | **ICC** |
| --- | --- | --- | --- |
| Vessel diameter index |  |  |  |
| Chu et al. | 0.3145 | 2.0144 | 0.8170 |
| Reif et al. | 0.5652 | 2.7693 | 0.3990 |
| Tang et al. | 0.7583 | 3.1640 | 0.4780 |
| Our method | 0.4830 | 2.2983 | 0.7120 |
| Vessel perimeter index |  |  |  |
| Chu et al. | 0.0066 | 2.1513 | 0.6110 |
| Reif et al. | 0.0066 | 2.2570 | 0.6550 |
| Tang et al. | 0.0112 | 4.4359 | 0.6470 |
| Our method | 0.0132 | 5.1807 | 0.7180 |
| Vessel complexity |  |  |  |
| Chu et al. | 51.2068 | 3.4202 | 0.8740 |
| Reif et al. | 59.1075 | 4.0261 | 0.6620 |
| Tang et al. | 90.5993 | 7.9588 | 0.6310 |
| Our method | 121.7700 | 9.9502 | 0.7160 |
| Vessel density |  |  |  |
| Chu et al. | 0.0128 | 2.5145 | 0.9280 |
| Reif et al. | 0.0085 | 1.7971 | 0.5560 |
| Tang et al. | 0.0148 | 3.2174 | 0.7310 |
| Our method | 0.0110 | 2.5809 | 0.8680 |
| Vessel skeleton density |  |  |  |
| Chu et al. | 0.0085 | 2.7881 | 0.7960 |
| Reif et al. | 0.0064 | 2.9099 | 0.3470 |
| Tang et al. | 0.0062 | 3.4344 | 0.7980 |
| Our method | 0.0056 | 2.9784 | 0.8190 |
| Vessel skeleton fractal dimension |  |  |  |
| Chu et al. | 0.0010 | 0.0510 | 0.6770 |
| Reif et al. | 0.0011 | 0.0578 | 0.4000 |
| Tang et al. | 0.0012 | 0.0647 | 0.8040 |
| Our method | 0.0011 | 0.0614 | 0.7970 |
| Vessel skeleton density in 1mm circle |  |  |  |
| Chu et al. | 0.0272 | 12.6704 | 0.6220 |
| Reif et al. | 0.0181 | 10.5836 | 0.5890 |
| Tang et al. | 0.0121 | 10.5046 | 0.7970 |
| Our method | 0.0121 | 11.9340 | 0.8630 |
| **Vessel skeleton density in 2mm circle** |  |  |  |
| Chu et al. | 0.0168 | 5.9318 | 0.5510 |
| Reif et al. | 0.0099 | 4.6925 | 0.3190 |
| Tang et al. | 0.0126 | 7.5335 | 0.5690 |
| Our method | 0.0154 | 9.3064 | 0.6770 |
| **Vessel density in 1mm circle** |  |  |  |
| Chu et al. | 0.0369 | 11.4790 | 0.7590 |
| Reif et al. | 0.0291 | 9.1564 | 0.7720 |
| Tang et al. | 0.0243 | 9.5730 | 0.8580 |
| Our method | 0.0275 | 14.0316 | 0.8470 |
| **Vessel density in 2mm circle** |  |  |  |
| Chu et al. | 0.0309 | 6.8603 | 0.7000 |
| Reif et al. | 0.0189 | 4.3707 | 0.5040 |
| Tang et al. | 0.0286 | 7.1871 | 0.5760 |
| Our method | 0.0343 | 9.9463 | 0.7000 |
| **Non-perfusion** |  |  |  |
| Chu et al. | 0.0867 | 51.2204 | 0.4730 |
| Reif et al. | 0.0970 | 48.5092 | 0.4720 |
| Tang et al. | 0.1853 | 41.9203 | 0.6030 |
| Our method | 0.1410 | 33.8663 | 0.7420 |

Sw, within-subject standard deviation; CoV, Coefficient of variation; ICC, Intraclass correlation coefficient.

Reliability and repeatability analysis of OCTA metrics in the left eyes

|  | **Sw** | **CoV** | **ICC** |
| --- | --- | --- | --- |
| **Vessel diameter index** |  |  |  |
| Chu et al. | 0.2793 | 1.7854 | 0.8680 |
| Reif et al. | 0.4188 | 2.0500 | 0.6210 |
| Tang et al. | 0.7870 | 3.2715 | 0.4400 |
| Our method | 0.4060 | 1.9227 | 0.7150 |
| **Vessel perimeter index** |  |  |  |
| Chu et al. | 0.0066 | 2.1614 | 0.5570 |
| Reif et al. | 0.0057 | 1.9447 | 0.6100 |
| Tang et al. | 0.0106 | 4.2060 | 0.6060 |
| Our method | 0.0115 | 4.4601 | 0.6970 |
| **Vessel complexity** |  |  |  |
| Chu et al. | 54.9885 | 3.6979 | 0.8460 |
| Reif et al. | 59.4880 | 4.0411 | 0.6180 |
| Tang et al. | 86.8493 | 7.7013 | 0.6040 |
| Our method | 104.2155 | 8.4714 | 0.7190 |
| **Vessel density** |  |  |  |
| Chu et al. | 0.0756 | 14.6915 | 0.9300 |
| Reif et al. | 0.0066 | 1.3823 | 0.6280 |
| Tang et al. | 0.0152 | 3.2723 | 0.6810 |
| Our method | 0.0131 | 3.0451 | 0.8000 |
| **Vessel skeleton density** |  |  |  |
| Chu et al. | 0.0079 | 2.5558 | 0.8270 |
| Reif et al. | 0.0049 | 2.2609 | 0.6900 |
| Tang et al. | 0.0067 | 3.7051 | 0.7370 |
| Our method | 0.0056 | 2.9671 | 0.7840 |
| **Vessel skeleton fractal dimension** |  |  |  |
| Chu et al. | 0.0008 | 0.0447 | 0.7570 |
| Reif et al. | 0.0008 | 0.0448 | 0.7110 |
| Tang et al. | 0.0013 | 0.0690 | 0.7490 |
| Our method | 0.0011 | 0.0604 | 0.7360 |
| **Vessel skeleton density in 1mm circle** |  |  |  |
| Chu et al. | 0.0244 | 11.3751 | 0.6940 |
| Reif et al. | 0.0156 | 9.1148 | 0.7170 |
| Tang et al. | 0.0114 | 9.8342 | 0.8180 |
| Our method | 0.0130 | 12.8177 | 0.8380 |
| **Vessel skeleton density in 2mm circle** |  |  |  |
| Chu et al. | 0.0129 | 4.4991 | 0.6790 |
| Reif et al. | 0.0067 | 3.1467 | 0.6490 |
| Tang et al. | 0.0105 | 6.1913 | 0.5990 |
| Our method | 0.0142 | 8.3934 | 0.5540 |
| **Vessel density in 1mm circle** |  |  |  |
| Chu et al. | 0.0344 | 10.6542 | 0.7790 |
| Reif et al. | 0.0230 | 7.3157 | 0.8300 |
| Tang et al. | 0.0237 | 9.3259 | 0.8360 |
| Our method | 0.0270 | 13.7495 | 0.8100 |
| **Vessel density in 2mm circle** |  |  |  |
| Chu et al. | 0.0241 | 5.2842 | 0.7770 |
| Reif et al. | 0.0116 | 2.6673 | 0.6390 |
| Tang et al. | 0.0245 | 6.0538 | 0.5430 |
| Our method | 0.0305 | 8.6395 | 0.6030 |
| **Non-perfusion** |  |  |  |
| Chu et al. | 0.0565 | 33.4453 | 0.7370 |
| Reif et al. | 0.0501 | 26.0374 | 0.7960 |
| Tang et al. | 0.1353 | 32.7880 | 0.5620 |
| Our method | 0.0815 | 21.3451 | 0.7590 |

**Supplementary Table 3.** The relationship between OCTA metrics and systemic risk factors. See “Supplementary Table 3.xlsx” for detail.

**Supplementary Table 4.** The results of the Wilcox test for OCTA metrics in the diabetes group and healthy group.

| **OCTA metrics** | **P values** | **P values adjusted by FDR** |
| --- | --- | --- |
| Vessel perimeter index | 0.0000 | 0.0000 |
| Vessel complexity index | 0.0000 | 0.0000 |
| Non-perfusion area | 0.0000 | 0.0000 |
| Small vessel density | 0.0000 | 0.0000 |
| Vessel skeleton fractal dimension | 0.0000 | 0.0000 |
| Vessel diameter index | 0.0000 | 0.0000 |
| Vessel skeleton density in 2mm circle | 0.0000 | 0.0000 |
| Superior vessel density of macular in superficial layer | 0.0000 | 0.0000 |
| Small vessel skeleton density | 0.0000 | 0.0000 |
| Vessel skeleton density | 0.0000 | 0.0000 |
| Vessel density in 1mm circle | 0.0000 | 0.0000 |
| Vessel skeleton density in 1mm circle | 0.0000 | 0.0000 |
| Superior vessel density of macular in deep layer | 0.0000 | 0.0000 |
| FAZ perimeter | 0.0000 | 0.0000 |
| Mean thickness of RNFL layer centered in the macular | 0.0000 | 0.0000 |
| FAZ vertical diameter | 0.0000 | 0.0000 |
| FAZ circularity index | 0.0000 | 0.0000 |
| Optic disc area | 0.0000 | 0.0001 |
| Vessel density in 2mm circle | 0.0000 | 0.0001 |
| Disc horizontal diameter | 0.0000 | 0.0001 |
| Inferior vessel density of macular in deep layer | 0.0000 | 0.0001 |
| Disc vertical diameter | 0.0001 | 0.0002 |
| FAZ major axis diameter | 0.0001 | 0.0003 |
| Mean thickness of GCL+ layer centered in the macular | 0.0005 | 0.0011 |
| Mean thickness of RNFL layer centered in the optic disc | 0.0010 | 0.0021 |
| FAZ area | 0.0020 | 0.0041 |
| Optic cup area | 0.0024 | 0.0049 |
| Vertical diameter ratio of cup and disc | 0.0034 | 0.0065 |
| Center vessel density of macular in superficial layer | 0.0049 | 0.0091 |
| Mean thickness of retina layer centered in the optic disc | 0.0065 | 0.0117 |
| Mean thickness of GCL++ layer centered in the optic disc | 0.0066 | 0.0115 |
| Mean thickness of Choroid layer centered in the optic disc | 0.0076 | 0.0128 |
| FAZ minor axis diameter | 0.0159 | 0.0261 |
| Optic rim area | 0.0214 | 0.0340 |
| Big vessel density | 0.0338 | 0.0521 |
| Area ratio of cup and disc | 0.0356 | 0.0534 |
| Square root of area ratio of cup and disc | 0.0379 | 0.0553 |
| Nasal vessel density of macular in deep layer | 0.0380 | 0.0540 |
| FAZ horizontal diameter | 0.0523 | 0.0724 |
| Temporal vessel density of macular in deep layer | 0.0524 | 0.0707 |
| FAZ eccentricity | 0.0576 | 0.0759 |
| Inferior vessel density of macular in superficial layer | 0.1023 | 0.1315 |
| Center vessel density of macular in deep layer | 0.1799 | 0.2259 |
| Optic rim volume | 0.1840 | 0.2258 |
| Big vessel skeleton density | 0.2075 | 0.2490 |
| Mean thickness of Choroid layer centered in the macular | 0.2995 | 0.3516 |
| Optic cup volume | 0.3005 | 0.3453 |
| Mean thickness of retina layer centered in the macular | 0.3150 | 0.3543 |
| FAZ orientation | 0.3651 | 0.4023 |
| Temporal vessel density of macular in superficial layer | 0.4214 | 0.4551 |
| Nasal vessel density of macular in superficial layer | 0.5831 | 0.6174 |
| Mean thickness of GCL++ layer centered in the macular | 0.6400 | 0.6646 |
| Vessel density | 0.8013 | 0.8164 |
| Mean thickness of GCL+ layer centered in the optic disc | 0.8592 | 0.8592 |

**Supplementary Figure 1.** The flowchart of the selection of study participants.
