# Supplementary figures and images for: Association Between the Severity of Diabetic Retinopathy and Optical Coherence Tomography Angiography Metrics
Source: Front Endocrinol (Lausanne). 2021 Dec 10;12:777552. doi: 10.3389/fendo.2021.777552 (PMC8702651; doi:10.3389/fendo.2021.777552)

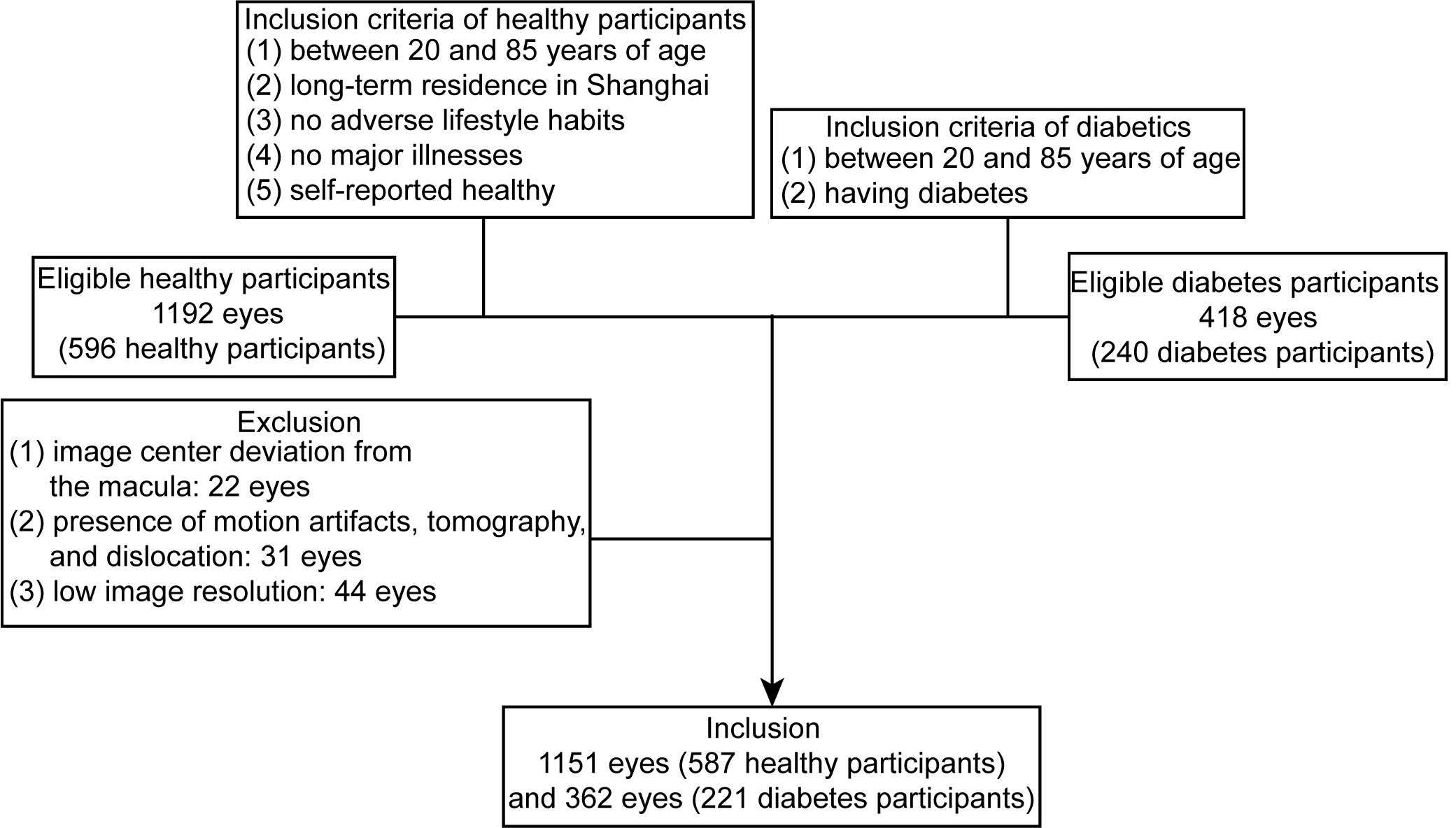

Supplement: Supplementary file 2 [file Image_1.tif]
